# Synergistic Effects of Orbital Shear Stress on *In Vitro* Growth and Osteogenic Differentiation of Human Alveolar Bone-Derived Mesenchymal Stem Cells

**DOI:** 10.1155/2014/316803

**Published:** 2014-01-14

**Authors:** Ki Taek Lim, Jin Hexiu, Jangho Kim, Hoon Seonwoo, Pill-Hoon Choung, Jong Hoon Chung

**Affiliations:** ^1^Department of Biosystems & Biomaterials Science and Engineering, Seoul National University, Seoul 151-921, Republic of Korea; ^2^Department of Oral and Maxillofacial Surgery and Dental Research Institute, School of Dentistry, Seoul National University, Seoul 110-744, Republic of Korea; ^3^Tooth Bioengineering National Research Laboratory of Post BK21, School of Dentistry, Seoul National University, Seoul 110-744, Republic of Korea; ^4^Research Institute for Agriculture and Life Sciences, Seoul National University, Seoul 151-921, Republic of Korea

## Abstract

Cellular behavior is dependent on a variety of physical cues required for normal tissue function. In order to mimic native tissue environments, human alveolar bone-derived mesenchymal stem cells (hABMSCs) were exposed to orbital shear stress (OSS) in a low-speed orbital shaker. The synergistic effects of OSS on proliferation and differentiation of hABMSCs were investigated. In particular, we induced the osteoblastic differentiation of hABMSCs cultured in the absence of OM by exposing hABMSCs to OSS (0.86–1.51 dyne/cm^2^). Activation of Cx43 was associated with exposure of hABMSCs to OSS. The viability of cells stimulated for 10, 30, 60, 120, and 180 min/day increased by approximately 10% compared with that of control. The OSS groups with stimulation of 10, 30, and 60 min/day had more intense mineralized nodules compared with the control group. In quantification of vascular endothelial growth factor (VEGF) and bone morphogenetic protein-2 (BMP-2) protein, VEGF protein levels under stimulation for 10, 60, and 180 min/day and BMP-2 levels under stimulation for 60, 120, and 180 min/day were significantly different compared with those of the control. In conclusion, the results indicated that exposing hABMSCs to OSS enhanced their differentiation and maturation.

## 1. Introduction

The stem cell is a complex microenvironment combining an extracellular matrix, cell-to-cell interactions, and other factors such as growth factors, physical factors, and various cytokines. Stem cells are exposed to high Ca^2+^ concentrations and a variety of autocrine, paracrine, and endocrine signals (extrinsic factors) and they are attached to the ECM through integrin receptors [1–10]. Many researchers have already reported an influence of cell growth and differentiation with the use of physical stimulators. Also, we have previously reported the *in vitro* osteogenic effects of cell stimulation on human alveolar bone-derived mesenchymal stem cells (hABMSCs) using a simple rocking culture method [11]. Thereby, we ascertained that the shear stress on hABMSCs could significantly enhance cell migration, proliferation, and differentiation. Our previous study motivated us to identify other methods for simple cell stimulation.

Thus, we designed orbital shear stress (OSS), which considered another possible cell stimulation method with the concept that flow patterns within intraoral fluid in the mouth are circular. There have been several studies of the effects of OSS on cellular behaviors [2–12]. Steady laminar flow can induce the expression of many genes and proteins in stem cells. The physical forces have profound effects on the cytoskeleton and extracellular matrix. These cellular components are essential in maintaining the integrity of stem cells. In particular, gap junctions are membrane channels that mediate the cell-to-cell movement of ions and small metabolites [5, 6, 13]. Some studies have reported that the Cx43 which is involved in gap junction channel activity in cells, including stem cells, might be induced by OSS to regulate cell growth and differentiation [6–10]. It has been suggested that the mode of cell-cell communication might be of particular importance in the skeleton, where various signals mediate gap junction communication and connexin biology in the bone [8–10, 14, 15]. Above all, one mechanism of cell-cell interaction is direct cell-cell communication via gap junctions, which are transmembrane channels that allow for the continuity of cytoplasm between communicating cells [13–15]. Cellular signaling occurs through distinct events: binding of stimuli secreted from neighboring cells or cell junctions and release in response to stimuli. Such signals affect cellular migration, growth, and differentiation [16–18].

The purpose of our study, therefore, was to investigate the synergistic effects of OSS on *in vitro* growth and osteogenic differentiation of hABMSCs for tissue engineering applications.

## 2. Materials and Methods

### 2.1. Cell Culture

hABMSCs were collected at the Intellectual Biointerface Engineering Center, Dental Research Institute, College of Dentistry, Seoul National University. hABMSCs were placed in 35 mm culture dishes at a density of 1.0 × 10^4^ cells/cm^2^ and cultured for 5 and 10 days. Cells were cultured in *α*-minimum essential medium (*α*-MEM) containing 10% fetal bovine serum (FBS, Welgene Inc., Republic of Korea) and 10 mM ascorbic acid (L-ascorbic acid) and antibiotics (Antibiotic-Antimycotic solution, Gibco) at 37°C in a humidified atmosphere of 5% CO_2_ (Steri-Cycle 370 Incubator, Thermo Fisher Scientific, USA). The cells were then incubated with osteogenic medium (100 nM dexamethasone, 50 *μ*g/mL of ascorbic acid, and 10 nM of *β*-glycerophosphate; Sigma) for 10 days. The induction culture medium was changed every second or third day. The proliferation and osteogenic differentiation of the cells were examined after exposure to each OSS.

### 2.2. Stimulation Treatment of OSS and Experimental Device

**Figure 1:**
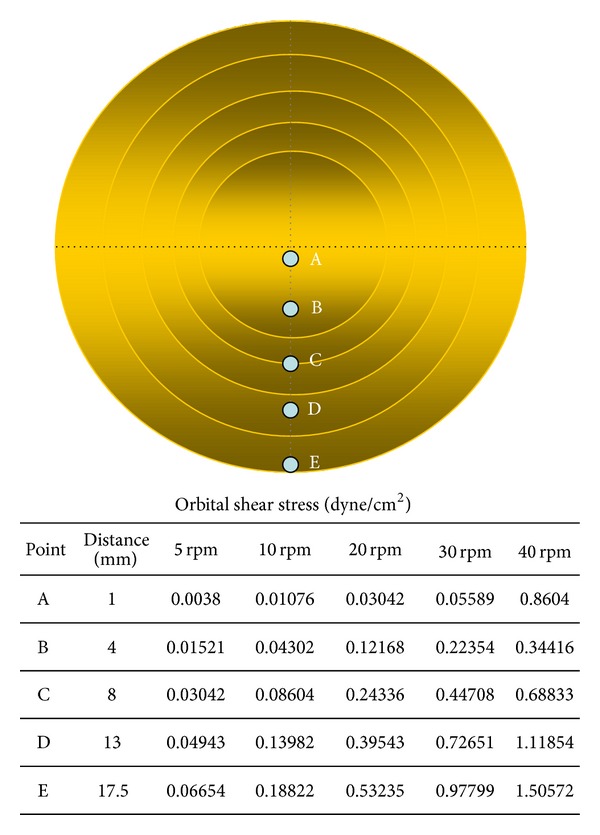
The schematic diagram with temporal points for calculating OSS values.

OSS was applied to confluent cell cultures using a low-speed orbital shaker (Benchmark Scientific, USA). The OSS was calculated using the following equation (1) [2]:

(1) $$\begin{array}{c} \tau_{w}=a\times \sqrt{\rho \times u\times {\left(2\times \pi \times f\right)}^{3}}, \end{array}$$

where *τ*_*w*_ is shear stress, *a* is the orbital radius of rotation of the shaker, *ρ* is the density of the culture medium, *μ* is the viscosity of the medium, and *f* is the frequency of rotation [2]. In this study, we calculated the values of shear stress at temporal points as shown in Figure 1 as 5, 10, 20, 30, and 40 rpm (revolutions per minute). The equation expresses constant magnitude of shear [17–19]. Figure 1 indicates temporal points for calculating values of OSS. The Reynolds number was calculated as *ωR* ^2^/*v*, where *ω* is the rotational speed of the orbital shaker, *R* is the radius of rotation of the orbital shaker (17.5 mm), and *v* is the kinematic viscosity (1.012 × 10^−6^ m^2^/s). hABMSCs were exposed to OSS (0.86–1.51 dyne/cm^2^) with plate on the orbital shaker (Reynolds number of 121). There were six treatment groups, stimulated for 10, 30, 60, 120, and 180 min/day.

### 2.3. Cell Viability, DNA Analysis, and *In Vitro* Cell Migration Assay

hABMSC proliferation was measured by WST-1 assay (EZ-Cytox Cell Viability Assay Kit, Daeillab Service Co., Ltd.). The formazan dye produced by viable cells was quantified by a multiwell spectrophotometer (Victor 3, Perkin Elmer, USA), measuring the absorbance of the dye solution at 460 nm. DNA concentration was quantified by fluorometry using the CyQUANT Cell Proliferation Assay Kit (Invitrogen), and the *λ* Fluorescence was measured using a Cytofluor II fluorescence multiwell plate reader with excitation of 485 nm and emission of 530 nm. *In vitro* cell migration was assessed by the CytoSelect Wound Healing Assay according to the manufacturer's protocols. Wound closure was measured by microscopy for up to 72 h, and photographs were taken. Cells were cultured with or without OSS, and cell morphology was observed by phase-contrast microscopy (Nikon TS100, Japan). hABMSCs were stimulated with exposure to OSS for 72 h, and the control was not exposed to OSS.

### 2.4. Measurement of Mineralized Nodule Formation

All cells except control cells were exposed to OSS for 10 days. Nodule formation was checked routinely by phase contrast microscopy. The presence of mineralized nodules (calcium deposition) was determined by staining with Alizarin red, as described [20]. The ethanol-fixed cells and matrix were stained for 1 h with 40 mM Alizarin red-S (pH 4.2) and extensively rinsed with water. After photography, the bound stain was eluted with 10% (wt/vol) cetylpyridinium chloride, and the Alizarin red staining in the samples was quantified by measuring absorbance at 544 nm (Victor 3, Perkin Elmer, USA). Cells were fixed with 4% (wt/vol) formaldehyde in PBS for 15 min. And the cells were incubated in 5% (wt/vol) silver nitrate (Sigma-Aldrich, USA) for 1 h under ultraviolet light condition, followed by incubation in 5% (wt/vol) sodium thiosulfate (Sigma-Aldrich, USA) for 5 min. Last, the wells were rinsed with distilled water twice and air-dried, and mineralization images were captured using an optical microscope.

### 2.5. Reverse Transcriptase-Polymerase Chain Reaction Analysis

Reverse transcriptase-polymerase chain reaction analysis (RT-PCR) was used to measure the expression of various osteogenic factors. After 10 days in OSS culture, total RNA was isolated with TRIzol reagent (Invitrogen) and used to synthesize cDNA using a first-strand cDNA synthesis kit (Invitrogen) according to the instructions of the manufacturer. The human primers used in this study are listed in Table 1. RNA was extracted from the cells 10 days after the addition of differentiation media. These extracts were subjected to RT-PCR analysis of Runx2 (runt-related transcription factor 2), COL1 (collagen type I), OCN (osteocalcin), OPN (osteopontin), SMAD1, and GAPDH (glyceraldehyde-3-phosphate dehydrogenase) as the positive control. The products were separated by electrophoresis on a 1% agarose gel (SeaKem ME; FMC Bioproducts) and visualized by ultraviolet-induced fluorescence. Expression levels of gene areas were measured using Image J 1.45s (National Institutes of Health).

### 2.6. Fluorescence Microscopy and Confocal Laser Scanning Analysis

Cells were washed in phosphate-buffered saline (PBS, Sigma-Aldrich, Milwaukee, WI, USA), fixed in a 4% paraformaldehyde solution (Sigma-Aldrich, Milwaukee, WI, USA) for 20 min, and permeabilized with 0.2% Triton X-100 (Sigma-Aldrich, WI, Milwaukee, USA) for 15 min. Cells were incubated with TRITC-conjugated phalloidin, antivinculin, its secondary antibody (Millipore Cat. no. AP124F), and DAPI (Millipore, Billerica, MA, USA) for 1 h to stain actin filaments, focal contacts, and nuclei, respectively. Cytoskeleton organization was visualized using an actin cytoskeleton and focal adhesion staining kit (FAK100; Millipore, Billerica, MA) according to the manufacturer's instruction. Cells were mounted in glycerol/buffer on a glass slide after extensive washing with PBS. Images of labeled cells were obtained using a fluorescence image restoration microscope (Applied Precision, USA).

To investigate specific proteins, cells were incubated with TRITC-conjugated phalloidin, antiosteocalcin, its secondary antibody (Cat. no. AB10911, Millipore), and DAPI (Millipore, Billerica, MA, USA) for 1 h to stain actin filaments, focal contracts, and nuclei, respectively. In addition, the major intermediate filament protein of the cells was visualized using an anti-Cx43 antibody (Cat. no. AB1728, Millipore) according to the manufacturer's protocol. Immunostaining with primary antibodies was used as a control, and at least two independent stainings were performed. Cells were mounted in glycerol/buffer on a glass slide after extensive washing with PBS. Images of labeled cells were obtained by a Confocal Laser Scanning Microscope (Carl Zeiss, LSM710).

### 2.7. ELISA Assay

To measure the levels of vascular endothelial growth factor (VEGF) and bone morphogenetic protein-2 (BMP-2), we used an ELISA kit with specific antibodies (Quantikine human VEGF and Quantikine human BMP-2 immunoassays, R&D Systems, USA). The culture supernatants were collected to quantify the levels of VEGF and BMP-2 produced from hABMSCs *in vitro *after 5 days. The assay protocol was performed according to the instructions of the manufacturer. Each sample was measured in triplicate.

### 2.8. Statistical Analysis

Statistical analysis was carried out using the SAS Statistical Analysis System for Windows v9.3 (SAS Institute, Inc., Cary, NC, USA). Statistical significance between control and treatment groups was compared with *t*-test, two-way ANOVA, and Duncan's multiple range tests at *P < 0.05. The data are reported as the mean ± standard deviation.

## 3. Results and Discussion

### 3.1. Cell Viability and Growth Are Enhanced by OSS

**Figure 2:**
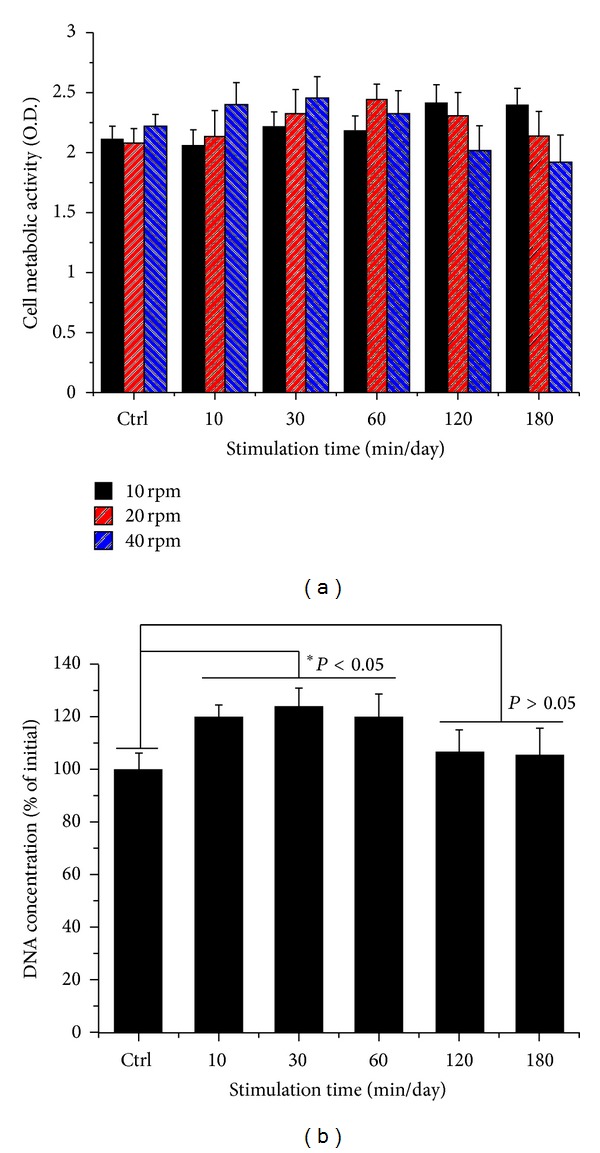
Cell metabolic viability as optical density of hABMSCs measured using WST-1 (a). DNA concentration as percent of initial hABMSCs measured using CyQUANT Cell Proliferation Assay Kit (b) (*n* = 3). Overhead brackets with asterisks indicate significant differences between groups.

**Figure 3:**
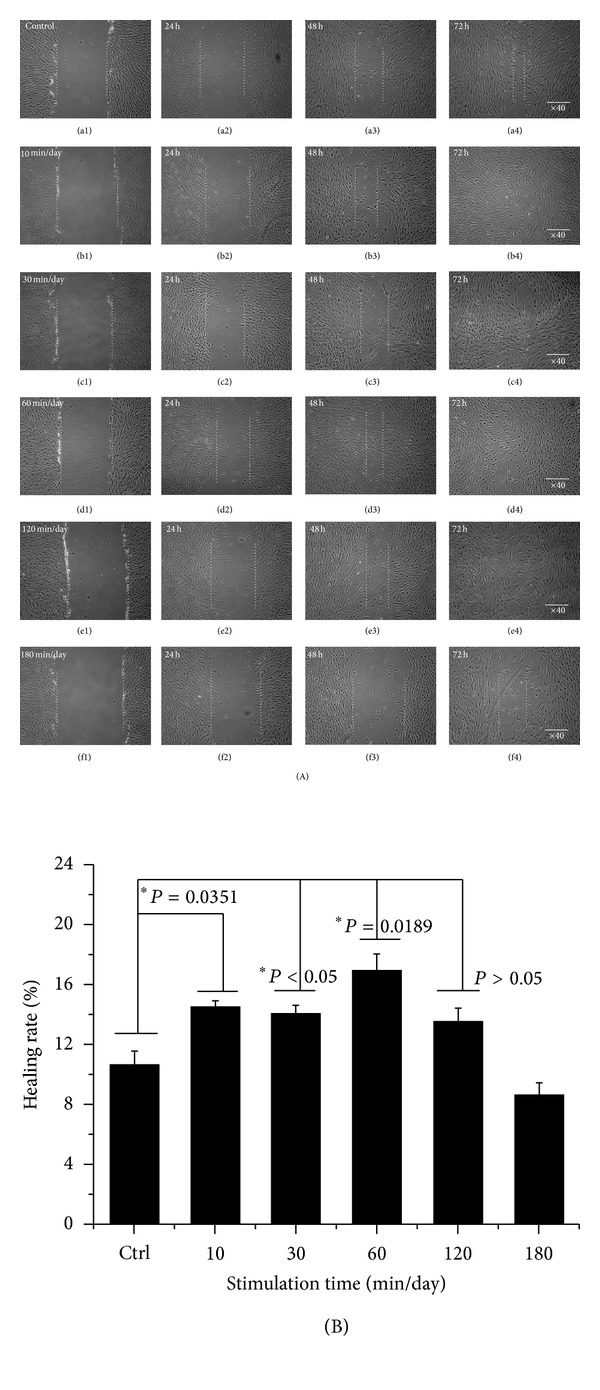
*In vitro* cell migration as representative optical microscopic images with OSS groups compared to static culture (A), indicating that stimulation groups exposed at 10, 30, and 60 min/day were significantly different (*P < 0.05) among groups (B) (*n* = 3).

Cell metabolic viability of hABMSCs was measured using optical density and WST-1 according to Figure 1. The cell viability of the 40 rpm group when exposed at 10 min/day increased more than 10% over those of 10 and 20 rpm groups (Figure 2(a)). DNA concentration (Figure 2(b)) as a percentage of initial hABMSC measured using the CyQuant cell proliferation with OSS stimulation (40 rpm). Specifically, we observed that 40 rpm and OSS stimulation of 10, 30, and 60 min/day induced greater cell metabolic activity. OSS groups had higher cell metabolic viability than control group. We have indicated that OSS in short term stimulated the cell growth and proliferation in vitro whereas hABMSCs proliferation was associated with decrease with exposure to laminar shear stress for long term. The *in vitro* hABMSCs migration result was shown in Figure 3. The *in vitro* cell migration shown in the optical microscopic images (A) showed that the difference between OSS and static culture groups was significant, and the OSS groups exposed at 10, 30, and 60 min/day also showed significant differences (*P < 0.05) (B). Based on the cell growth, migration assay, and DNA proliferation, hABMSCs proliferated significantly (about 20%) under OSS condition of 10 and 30 min/day when compared with that of control. We could consider that OSS does produce laminar shear stress on the cell-seeded culture dish, which is related to increased proliferation.

### 3.2. Enhanced Gap Junction (Cx43) and OCN in the Absence of Osteogenic Media (OM)

**Figure 4:**
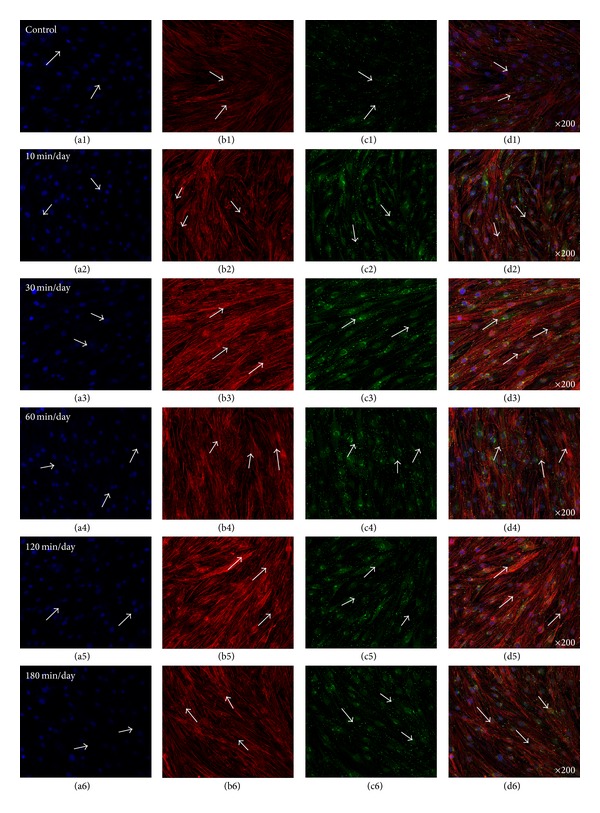
Representative optical fluorescence microscopy images of hABMSCs cultured for 5 days in static conditions (a1–d1) or at 10 min/day (a2–d2), 30 min/day (a3–d3), 60 min/day (a4–d4), 120 min/day (a5–d5), and 180 min/day (a6–d6) by OSS without OM; cell nuclei (a1–a6), actin filaments (b1–b6), gap junction (Cx43, c1–c6), and merged images (d1–d6) of the fluorescence stains. Fluorescence images showed more intense observation in OSS groups without OM compared to those in control (arrows: cell direction).

**Figure 5:**
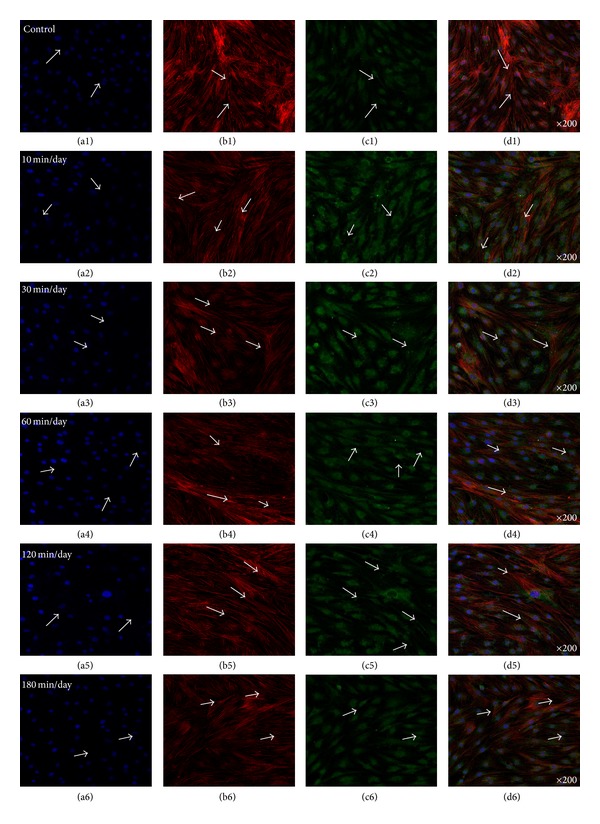
Representative optical fluorescence microscopy images of hABMSCs cultured for 5 days in static conditions (a1–d1) or at 10 min/day (a2–d2), 30 min/day (a3–d3), 60 min/day (a4–d4), 120 min/day (a5–d5), and 180 min/day (a6–d6) by OSS without OM; cell nuclei (a1–a6), actin filaments (b1–b6), OCN (c1–c6), and merged images (d1–d6) of the fluorescence stains. Fluorescence images showed more intense observation in OSS groups without OM compared to those of control.

Figure 4 showed representative confocal images of hABMSCs cultured for 5 days in static conditions (a1–d1) or at 10 min/day (a2–d2), 30 min/day (a3–d3), 60 min/day (a4–d4), 120 min/day (a5–d5), and 180 min/day (a6–d6) by OSS in the absence of OM; cell nuclei (a1–a6), actin filaments (b1–b6), gap junctions (Cx43, c1–c6), and merged images (d1–d6) of the fluorescence stains. The Cx43 indicated more intense staining in OSS groups in the absence of OM compared with the control. Gap junction communication is important in bone cells [21], where the channels are involved in mechanical transmission [22–24], induction of cytokines in osteoblasts [25], and coordination of hormonal responses [26, 27]. In osteoblast-like cells *in vitro*, Cx43 is the dominant connexin subtype and likely plays an important role in normal skeletal development [28–30]. Many studies have demonstrated a mutual relationship between cell growth and the expression of tissue-specific genes during mineralization [31–33]. In this respect, we could assure that gap junction (Cx43) was accelerated by OSS compared with that of control in the absence of OM.  Figure 5 presented representative optical fluorescence microscopy images of hABMSCs cultured for 5 days in static conditions (a1–d1) or at 10 min/day (a2–d2), 30 min/day (a3–d3), 60 min/day (a4–d4), 120 min/day (a5–d5), and 180 min/day (a6–d6) by OSS with OM; cell nuclei (a1–a6), actin filaments (b1–b6), OCN (osteocalcin, c1–c6), and merged images (d1–d6) of the fluorescence stains. Fluorescence images of OCN ascertained that gap junction (Cx43) was affected by OSS even in the absence of OM compared to those of the control, suggesting that the shear stress stimulates the cells mechanically and alters cellular functions.

### 3.3. Gene Expression of Osteoblastic Differentiation Markers

**Figure 6:**
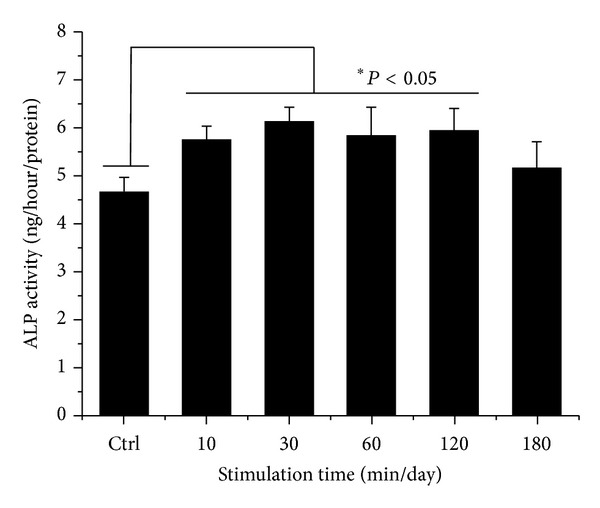
ALP activity cultured in different types of hABMSCs exposed with OSS for 7 days. The groups exposed at 10, 30, 60, and 120 min/day were significantly different among groups (*n* = 3).

**Figure 7:**
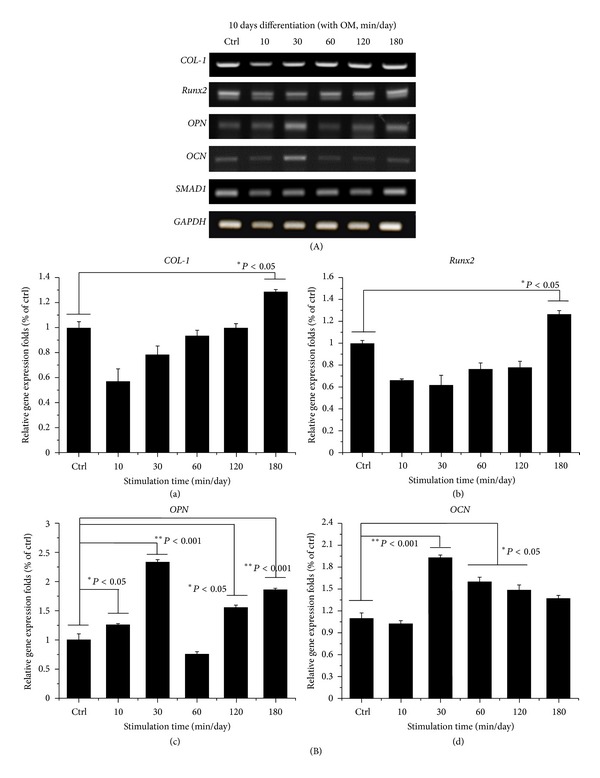
RT-PCR analysis of cell cultures between stimulus conditions (from 10 min/day to 180 min/day) and static culture for 10 days (A). RNA was extracted from the cell cultures at 10 days after the addition of differentiation media. These extracts were subjected to RT-PCR analysis with Runx2, COL1, OCN, OPN, SMAD1, and GAPDH as the positive control. Expression levels (B) of COL-1 (a), Runx2 (b), OPN (c), and OCN (d) at 10 days were significantly higher in OSS stimulus conditions on cells than those in control. OSS groups exposed at 10, 30, 60, and 120 min/day were significantly different (*P < 0.05 and **P < 0.001) among groups.

We investigated alkaline phosphatase activity (ALP) of hABMSCs stimulated with OSS for 7 days (Figure 6). To induce osteoblast differentiation in MSCs, the culture medium was supplemented with osteogenic agents, including L-ascorbic acid, *β*-glycerophosphate, and dexamethasone [34–37]. L-Ascorbic acid enhances collagen synthesis and upregulates adenosine triphosphatase and ALP activity, and *β*-glycerophosphate serves primarily as a source of inorganic phosphate ions [2, 12, 34–40]. The results of RT-PCR analysis of the cell cultures between stimulus conditions (from 10 min/day to 180 min/day) and static culture for 10 days (A) were shown in Figure 7. Expression of genes associated with the osteoblastic differentiation was examined using RT-PCR to investigate the effect of the stimulation with OSS on gene expression at 10 days. Expression levels (B) of COL-1 (a), Runx2 (b), OPN (c), and OCN (d) at 10 days were higher in OSS stimulation conditions on cells than those in control. Stimulation groups of 10, 30, 60, and 120 min/day were significantly different (*P < 0.05 and **P < 0.001) among groups. In particular, the 30 min/day group showed high expression levels of OPN and OCN.

### 3.4. Osteoinduction of hABMSCs by OSS in the Absence of OM

**Figure 8:**
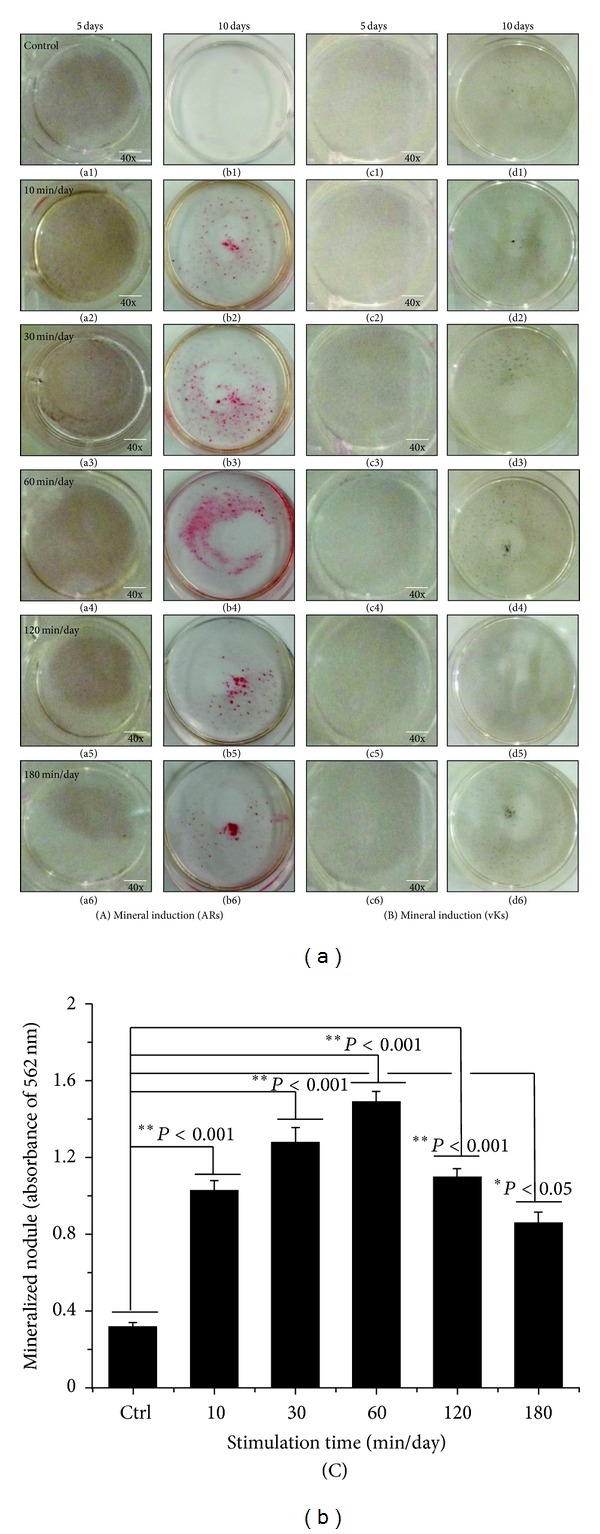
Representative optical microscopic images of hABMSCs after Alizarin red staining treatment with static condition (a1, b1) or at 10 min/day (a2, b2), 30 min/day (a3, b3), 60 min/day (a4, b4), 120 min/day (a5, b5), and 180 min/day (a6, b6) by OSS treatment in the absence of OM on 5 days and 10 days, respectively. OSS induction groups at 10, 30, and 60 min/day were intense compared to those of control (white arrows: mineral nodules stained in red). Representative microscopic images of hABMSCs after Von-Kossa staining with static condition (c1, d1) or at 10 min/day (c2, d2), 30 min/day (c3, d3), 60 min/day (c4, d4), 120 min/day (c5, d5), and 180 min/day (c6, d6) by OSS treatment in the absence of OM. Mineralized nodule as optical density measured after destaining treatment (C). OSS induction exposed at all of the groups was significantly different (*P < 0.05 and **P < 0.001) among groups (*n* = 3, bar = 1 mm).

Figure 8 indicated representative optical microscopic images of osteoinduction of hABMSCs after Alizarin red staining of cells treated with static conditions (a1, b1) or by stimulation for 10 min/day (a2, b2), 30 min/day (a3, b3), 60 min/day (a4, b4), 120 min/day (a5, b5), and 180 min/day (a6, b6) on 5 days or 10 days, respectively. The cells induced with OSS treatment during 10, 30, and 60 min/day were intense compared with those of control. Representative microscopic images after von-Kossa staining are also shown (Figure 8) for static condition (c1, d1) or for stimulation for 10 min/day (c2, d2), 30 min/day (c3, d3), 60 min/day (c4, d4), 120 min/day (c5, d5), and 180 min/day (c6, d6). The cells stimulated with OSS (C) showed significant differences in osteoinduction (*P < 0.05 and **P < 0.001) among groups. Interestingly, mineral induction via OSS indicated the cells were moving outwards. We considered that one of the migration roles of hABMSCs used in this study could be controlled to the desired migration direction as well as external force on cells. Ultimately, osteogenic differentiation promotion on hABMSCs was induced by the simple orbital shear shaker as physical cues of the microenvironment.

### 3.5. Effects of OSS Induction with OM on Osteogenic Differentiation

**Figure 9:**
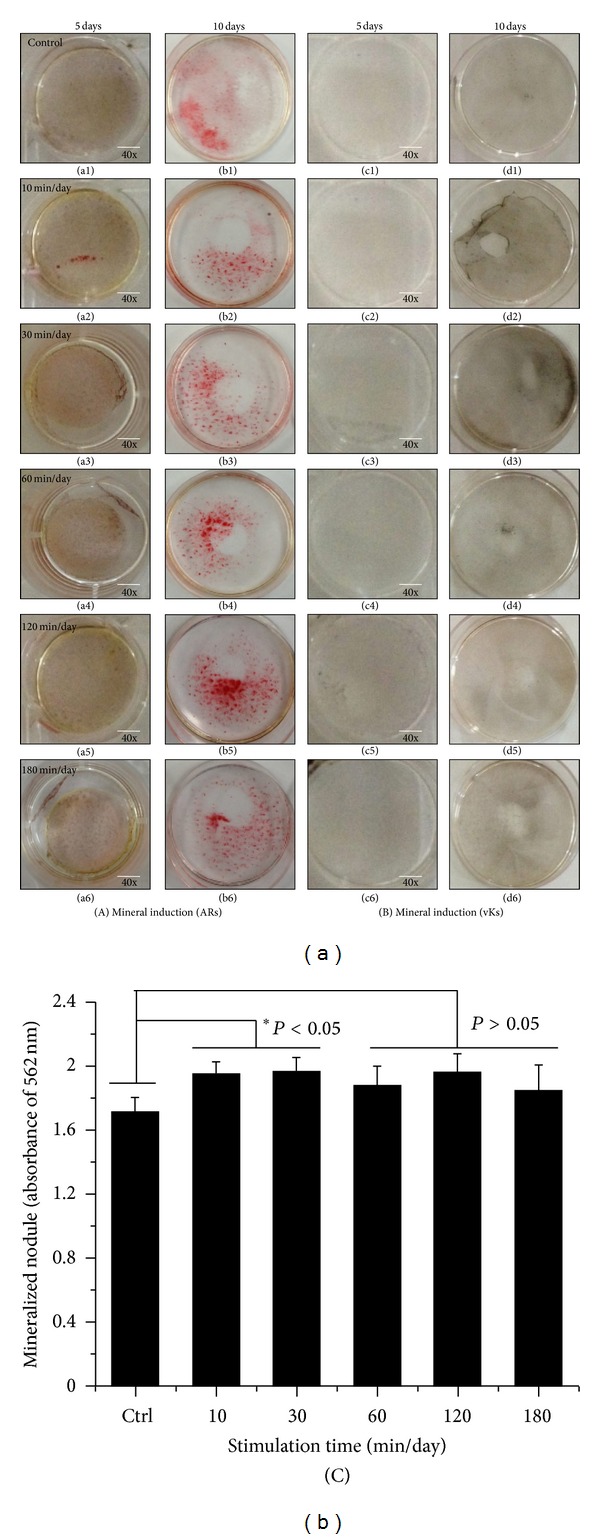
Representative optical microscopic images of hABMSCs after Alizarin red staining treatment with static condition (a1, b1) or at 10 min/day (a2, b2), 30 min/day (a3, b3), 60 min/day (a4, b4), 120 min/day (a5, b5), and 180 min/day (a6, b6) by OSS treatment with OM on 5 days and 10 days, respectively. OSS groups of 10, 30, and 60 min/day were intense compared to those of control. Representative microscopic images of hABMSCs after Von-Kossa staining with static condition (c1, d1) or at 10 min/day (c2, d2), 30 min/day (c3, d3), 60 min/day (c4, d4), 120 min/day (c5, d5), and 180 min/day (c6, d6) by OSS treatment with OM. Mineralized nodule as optical density measured after destaining treatment (C). OSS induction exposed at 10, 30, and 120 min/day groups was significantly different (*P < 0.05) among groups (*n* = 3, bar = 1 mm).

Figure 9 shows representative optical microscopic images of osteogenic differentiation of hABMSCs after Alizarin red staining of cells treated with static conditions (a1, b1) or by stimulation for 10 min/day (a2, b2), 30 min/day (a3, b3), 60 min/day (a4, b4), 120 min/day (a5, b5), and 180 min/day (a6, b6) on 5 or 10 days, respectively. Cells treated with OSS for 10, 30, and 60 min/day were intense compared to those of control. Representative images of von Kossa-stained hABMSCs treated with static conditions (c1, d1) or by stimulation for 10 min/day (c2, d2), 30 min/day (c3, d3), 60 min/day (c4, d4), 120 min/day (c5, d5), and 180 min/day (c6, d6) were also shown. Cells treated with OSS induction for 10, 30, and 120 min/day showed significant differences (C, *P < 0.05) among groups.

Several studies have shown effects of OSS on *in vitro* growth of cells and experimental apparatus that can provide quantifiable shear stress, involving inducing a rotating flow. In particular, endothelial cells could experience shear exerted by the flow of blood, causing them to become aligned and elongated with the direction of flow and to undergo other biochemical changes [2, 41]. More importantly, orbital shakers provide oscillatory flow, somewhat like the pulsing fluid movement in the human vasculature system [41–43]. In the biopharmaceutical development, agitation varies from simple mixing of components to increasing mass transfer, to deliberate introduction of agitation-related stresses and to accelerate protein degradation in screening experiments [44]. Based on these facts, we ascertained that the cell stimulation could mediate a strong effect on cell proliferation and differentiation.

### 3.6. Analysis of Gap Junction (Cx43) and OCN with OM

**Figure 10:**
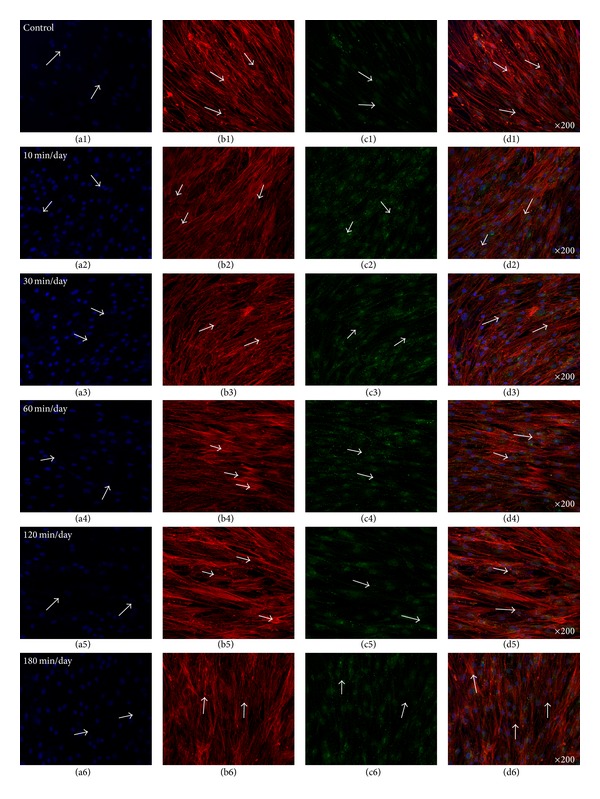
Representative optical fluorescence microscopy images of hABMSCs cultured for 5 days in static conditions (a1–d1) or at 10 min/day (a2–d2), 30 min/day (a3–d3), 60 min/day (a4–d4), 120 min/day (a5–d5), and 180 min/day (a6–d6) by OSS with OM; cell nuclei (a1–a6), actin filaments (b1–b6), gap junction (Cx43, c1–c6), and merged images (d1–d6) of the fluorescence stains. Fluorescence images showed more intense observation at OSS groups compared to those of control (arrows: cell direction).

Figure 10 shows representative optical fluorescence images of hABMSCs cultured for 5 days under static condition (a1–d1) or under stimulation for 10 min/day (a2–d2), 30 min/day (a3–d3), 60 min/day (a4–d4), 120 min/day (a5–d5), and 180 min/day (a6–d6) by OSS with OM; cell nuclei (a1–a6), actin filaments (b1–b6), gap junction (Cx43, c1–c6), and merged images (d1–d6) of the fluorescence stains. Fluorescence images indicated more intense staining in cells treated with OSS compared with those of controls. Interestingly, the gap junction (Cx43) fluorescence stains of cells cultured with PM (proliferation media) were more strong compared with cells cultured with OM.

**Figure 11:**
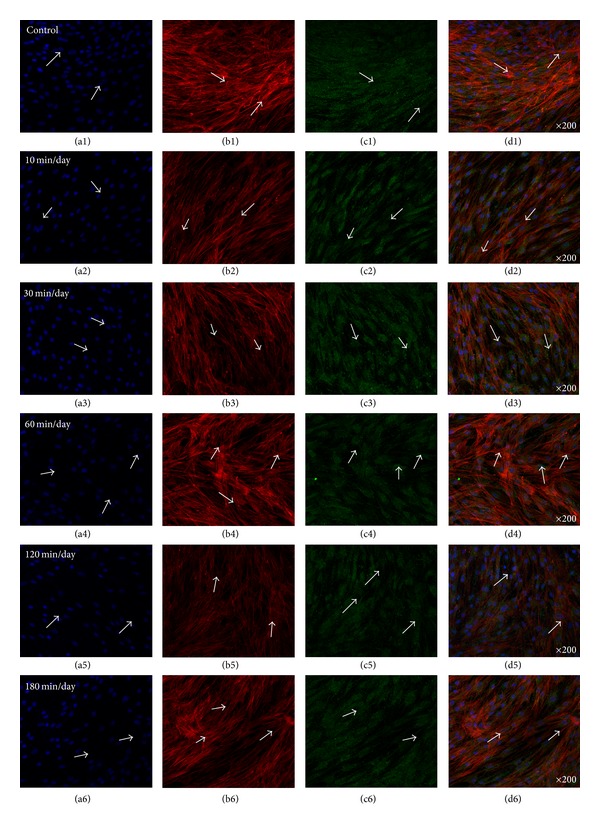
Representative optical fluorescence microscopy images of hABMSCs cultured for 5 days in static conditions (a1–d1) or at 10 min/day (a2–d2), 30 min/day (a3–d3), 60 min/day (a4–d4), 120 min/day (a5–d5), and 180 min/day (a6–d6) by OSS with OM; cell nuclei (a1–a6), actin filaments (b1–b6), (OCN, c1–c6), and merged images (d1–d6) of the fluorescence stains. Fluorescence images showed more intense observation in OSS groups with OM compared to those of control (arrows: cell direction).

Figure 11 demonstrates representative optical fluorescence images of hABMSCs cultured for 5 days under static conditions (a1–d1) or under stimulation for 10 min/day (a2–d2), 30 min/day (a3–d3), 60 min/day (a4–d4), 120 min/day (a5–d5), and 180 min/day (a6–d6) by OSS with OM; cell nuclei (a1–a6), actin filaments (b1–b6), OCN (osteocalcin, c1–c6), and merged images (d1–d6) of the fluorescence stains. The fluorescence images presented more deep staining in the cells stimulated by OSS with OM compared to those of control.

Gap junction intercellular communication is the most direct way of achieving such signaling, and gap junction communication through connexin-mediated junctions, in particular connexin 43 (Cx43), plays a major role bone development [45]. Given the important role of Cx43 in controlling development and differentiation, especially in bone cells, controlling the expression of Cx43 may provide control over cell-to-cell communication and may help overcome some of the challenges in craniofacial tissue engineering [45–47].

Connexins play a major role in response to many mechanical, electrical, chemical, and hormonal stimuli and help regulate cell homeostasis as well as calcium signaling and differentiation [26, 48, 49]. Therefore, controlling fluid flow like OSS can also potentially induce the opening of Cx43 hemichannels in osteocytes and other bone cells allowing for enhanced cell-cell communication and bone formation [50–52]. The major premise of functional tissue engineering is to provide physical cues to cells as a means of enhancing proliferation, differentiation, and tissue formation. Physical stimulation of cells in monolayer enhances gap junction function [23, 49, 50, 53, 54]. Thus, the mechanisms with relation to the enhanced tissue regeneration that are subjected to physical stimulation may be gap junction mediated [55].

### 3.7. Quantitative Analysis of BMP-2 and VEGF Proteins

**Figure 12:**
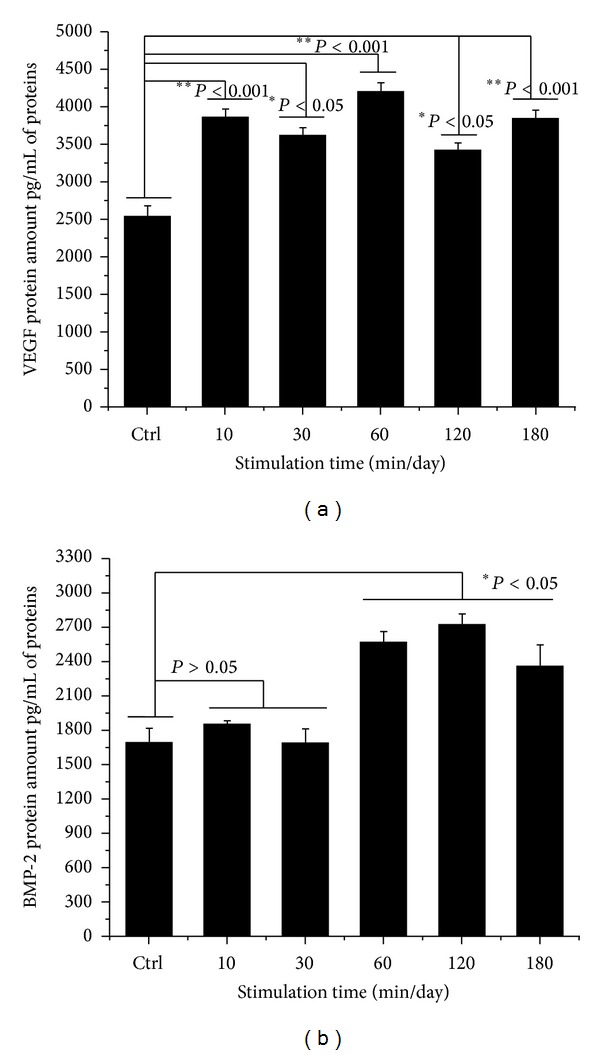
Quantitative analysis of VEGF and BMP-2 proteins was performed with conditioned medium between OSS treatment and control group. VEGF protein of OSS group was significantly different (30 and 120 min/day; *P < 0.05, 10, 60, and 180 min/day; **P < 0.001). BMP-2 protein of OSS induction group also showed significant differences (60, 120, and 180 min/day; *P < 0.05). Overhead brackets with asterisks indicated significant differences between groups.

Quantitative analysis of VEGF and BMP-2 proteins was performed with conditioned medium. VEGF protein of cells in the OSS induction group showed significant differences, as shown in Figure 12 (30 and 120 min/day; *P < 0.05, 10, 60, and 180 min/day; **P < 0.001). BMP-2 protein in the OSS induction group also indicated significant differences (60, 120, and 180 min/day; *P < 0.05). The interaction between VEGF and BMP-2 is dependent on the ratios of angiogenic and osteogenic factors. Osteogenic factors such as BMP-2 can stimulate osteoblasts, and VEGF can modulate vascularization [56–58].

## 4. Conclusions

In this study, we investigated the synergistic effects of OSS on *in vitro* growth and osteogenic differentiation of hABMSCs. The results indicated that OSS stimulation treatment has an important effect on the activation of mechanotransduction. Cell viability stimulated for 10, 30, and 60 min/day increased by about 10% compared with that of the control. We also found an effect of OSS on the osteogenic differentiation of hABMSCs with OM and without OM, respectively. The OSS groups with OM and without OM that underwent stimulation for 10, 30, and 60 min/day showed more intense staining compared with the control. We also quantified VEGF and BMP-2 protein expression levels after stimulation for 10, 60, and 180 min/day and found that VEGF protein levels and BMP-2 protein levels after 60, 120, and 180 min/day were significantly different from levels measured in the control. In conclusion, this study showed that exposing hABMSCs to OSS stimulation enhanced cell differentiation and maturation.

## Figures and Tables

**Table 1:** Human primer sequences.

Gene	Sequence (5′-3′)	Acc. no.	PCR cycles	Product size
GAPDH	ACCACAGTCCATGCCATCA	NM_002046	22	452
	TCCACCACCCTGTTGCTGT			

COL-1	CTGACCTTCCTGCGCCTGATGTCC	XM_012651	23	300
	GTCTGGGGCACCAACGTCCAAGGG			

RUNX2	CGCATTCCTCATCCCAGTAT	NM_001015051	30	462
	GACTGGCGGGGTGTAAGTAA			

OPN	CCCACAGACCCTTCCAAGTA	J04765	29	279
	ACACTATCACCTCGGCCATC			

OCN	GTGCAGAGTCCAGCAAAGGT	X53698	29	175
	TCAGCCAACTCGTCACAGTC			

SMAD1	CAACGCCACTTTTCCAGATT	U59423	30	313
	GCACCAGTGTTTTGGTTCCT			

ALP: alkaline phosphatase; GAPDH: glyceraldehyde-3-phosphate dehydrogenase; COL-I: collagen type I; RUNX2: runt-related transcription factor 2; OPN: osteopontin; OCN: osteocalcin; SMAD1: SMAD family member 1.

## References

[1] Chakraborty A (2011). *Fluid dynamic analysis of flow in orbiting dishes and the effects of flow on shear stress and endothelial cellular responses [Dissertation]*.

[2] Dardik A, Chen L, Frattini J (2005). Differential effects of orbital and laminar shear stress on endothelial cells. *Journal of Vascular Surgery*.

[3] Trump LR (2011). *Cell-cell Communication in three dimensional microenvironments [Dissertation]*.

[4] Al-Sukhun J, Lindqvist C, Kontio R (2006). Modelling of orbital deformation using finite-element analysis. *Journal of the Royal Society Interface*.

[5] Lo CW (2000). Role of gap junctions in cardiac conduction and development: insights from the connexin knockout mice. *Circulation Research*.

[6] Potter CM, Lundberg MH, Harrington LS (2011). Role of shear stress in endothelial cell morphology and expression of cyclooxygenase isoforms. *Arteriosclerosis, Thrombosis, and Vascular Biology*.

[7] Asada H, Paszkowiak J, Teso D (2005). Sustained orbital shear stress stimulates smooth muscle cell proliferation via the extracellular signal-regulated protein kinase 1/2 pathway. *Journal of Vascular Surgery*.

[8] Yamane T, Mitsumata M, Yamaguchi N (2010). Laminar high shear stress up-regulates type IV collagen synthesis and down-regulates MMP-2 secretion in endothelium. A quantitative analysis. *Cell and Tissue Research*.

[9] Gramsch B, Gabriel H-D, Wiemann M (2001). Enhancement of connexin 43 expression increases proliferation and differentiation of an osteoblast-like cell line. *Experimental Cell Research*.

[10] Civitelli R (2008). Cell-cell communication in the osteoblast/osteocyte lineage. *Archives of Biochemistry and Biophysics*.

[11] Lim KT, Kim J, Seonwoo H (2013). Enhanced osteogenesis of human alveolar bone-derived mesenchymal stem cells for tooth tissue engineering using fluid shear stress in a rocking culture method. *Tissue Engineering C*.

[12] Holtorf HL, Jansen JA, Mikos AG (2005). Flow perfusion culture induces the osteoblastic differentiation of marrow stromal cell-scaffold constructs in the absence of dexamethasone. *Journal of Biomedical Materials Research A*.

[13] Goodenough DA, Goliger JA, Paul DL (1996). Connexins, connexons, and intercellular communication. *Annual Review of Biochemistry*.

[14] Grellier M, Bordenave L, Amédée J (2009). Cell-to-cell communication between osteogenic and endothelial lineages: implications for tissue engineering. *Trends in Biotechnology*.

[15] Paul DL (1995). New functions for gap junctions. *Current Opinion in Cell Biology*.

[16] Rossello RA, Kohn DH (2010). Cell communication and tissue engineering. *Communicative and Integrative Biology*.

[17] Ley K, Lundgren E, Berger E, Arfors K-E (1989). Shear-dependent inhibition of granulocyte adhesion to cultured endothelium by dextran sulfate. *Blood*.

[18] Berson RE, Purcell MR, Sharp MK (2008). Computationally determined shear on cells grown in orbiting culture dishes. *Advances in Experimental Medicine and Biology*.

[19] Salek MM, Sattari P, Martinuzzi RJ (2012). Analysis of fluid flow and wall shear stress patterns inside partially filled agitated culture well plates. *Annals of Biomedical Engineering*.

[20] Williams DC, Boder GB, Toomey RE, Paul DC, Hillman CC, King KL (1980). Mineralization and metabolic response in serially passaged adult rat bone cells. *Calcified Tissue International*.

[21] Doty SB (1981). Morphological evidence of gap junctions between bone cells. *Calcified Tissue International*.

[22] Bowman NN, Donahue HJ, Ehrlich HP (1998). Gap junctional intercellular communication contributes to the contraction of rat osteoblast populated collagen lattices. *Journal of Bone and Mineral Research*.

[23] Donahue HJ (1998). Gap junctional intercellular communication in bone: a cellular basis for the mechanostat set point. *Calcified Tissue International*.

[24] Duncan RL, Turner CH (1995). Mechanotransduction and the functional response of bone to mechanical strain. *Calcified Tissue International*.

[25] Tanaka Y, Morimoto I, Nakano Y (1995). Osteoblasts are regulated by the cellular adhesion through ICAM-1 and VCAM-1. *Journal of Bone and Mineral Research*.

[26] Civitelli R, Ziambaras K, Warlow PM (1998). Regulation of connexin43 expression and function by prostaglandin E2 (PGE2) and parathyroid hormone (PTH) in osteoblastic cells. *Journal of Cellular Biochemistry*.

[27] Molen MAV, Rubin CT, McLeod KJ, McCauley LK, Donahue HJ (1996). Gap junctional intercellular communication contributes to hormonal responsiveness in osteoblastic networks. *Journal of Biological Chemistry*.

[28] Civitelli R, Beyer EC, Warlow PM, Robertson AJ, Geist ST, Steinberg TH (1993). Connexin43 mediates direct intercellular communication in human osteoblastic cell networks. *Journal of Clinical Investigation*.

[29] Donahue HJ, McLeod KJ, Rubin CT (1995). Cell-to-cell communication in osteoblastic networks: cell line-dependent hormonal regulation of gap junction function. *Journal of Bone and Mineral Research*.

[30] Schirrmacher K, Schmitz I, Winterhager E (1992). Characterization of gap junctions between osteoblast-like cells in culture. *Calcified Tissue International*.

[31] Lecanda F, Towler DA, Ziambaras K (1998). Gap junctional communication modulates gene expression in osteoblastic cells. *Molecular Biology of the Cell*.

[32] Stein GS, Lian JB, Owen TA (1990). Relationship of cell growth to the regulation of tissue-specific gene expression during osteoblast differentiation. *The FASEB Journal*.

[33] Aubin JE, Lui F, Malaval L, Gupta AK (1995). Osteoblast and chondroblast differentiation. *Bone*.

[34] Maniatopoulos C, Sodek J, Melcher AH (1988). Bone formation *in vitro* by stromal cells obtained from bone marrow of young adult rats. *Cell and Tissue Research*.

[35] Ter Brugge PJ, Jansen JA (2002). *In vitro* osteogenic differentiation of rat bone marrow cells subcultured with and without dexamethasone. *Tissue Engineering*.

[36] Rickard DJ, Sullivan TA, Shenker BJ, Leboy PS, Kazhdan I (1994). Induction of rapid osteoblast differentiation in rat bone marrow stromal cell cultures by dexamethasone and BMP-2. *Developmental Biology*.

[37] Peter SJ, Liang CR, Kim DJ, Widmer MS, Mikos AG (1998). Osteoblastic phenotype of rat marrow stromal cells cultured in the presence of dexamethasone, glycerolphosphate, and L-ascorbic acid. *Journal of Cellular Biochemistry*.

[38] Terkeltaub RA (2001). Inorganic pyrophosphate generation and disposition in pathophysiology. *The American Journal of Physiology*.

[39] Atmani H, Audrain C, Mercier L, Chappard D, Basle MF (2002). Phenotypic effects of continuous or discontinuous treatment with dexamethasone and/or calcitriol on osteoblasts differentiated from rat bone marrow stromal cells. *Journal of Cellular Biochemistry*.

[40] Porter RM, Huckle WR, Goldstein AS (2003). Effect of dexamethasone withdrawal on osteoblastic differentiation of bone marrow stromal cells. *Journal of Cellular Biochemistry*.

[41] Rochier A, Nixon A, Yamashita N (2011). Laminar shear, but not orbital shear, has a synergistic effect with thrombin stimulation on tissue factor expression in human umbilical vein endothelial cells. *Journal of Vascular Surgery*.

[42] Chakraborty A, Chakraborty S, Jala VR, Haribabu B, Sharp MK, Berson RE (2012). Effects of biaxial oscillatory shear stress on endothelial cell proliferation and morphology. *Biotechnology and Bioengineering*.

[43] Thomas JM, Chakraborty A, Sharp MK, Berson RE (2011). Spatial and temporal resolution of shear in an orbiting petri dish. *Biotechnology Progress*.

[44] Bai G, Bee JS, Biddlecombe JG, Chen Q, Leach WT (2012). Computational fluid dynamics (CFD) insights into agitation stress methods in biopharmaceutical development. *International Journal of Pharmaceutics*.

[45] Rossello RA, Kohn DH (2009). Gap junction intercellular communication: a review of a potential platform to modulate craniofacial tissue engineering. *Journal of Biomedical Materials Research B*.

[46] Bevans CG, Kordel M, Rhee SK, Harris AL (1998). Isoform composition of connexin channels determines selectivity among second messengers and uncharged molecules. *Journal of Biological Chemistry*.

[47] Vaney DI, Nelson JC, Pow DV (1998). Neurotransmitter coupling through gap junctions in the retina. *Journal of Neuroscience*.

[48] Wyatt LE, Chung CY, Carlsen B (2001). Bone morphogenetic protein-2 (BMP-2) and transforming growth factor-beta1 (TGF-beta1) alter connexin 43 phosphorylation in MC3T3-E1 Cells. *BMC Cell Biology*.

[49] Jiang JX, Siller-Jackson AJ, Burra S (2007). Roles of gap junctions and hemichannels in bone cell functions and in signal transmission of mechanical stress. *Frontiers in Bioscience*.

[50] Thi MM, Kojima T, Cowin SC, Weinbaum S, Spray DC (2003). Fluid shear stress remodels expression and function of junctional proteins in cultured bone cells. *The American Journal of Physiology*.

[51] Cherian PP, Siller-Jackson AJ, Gu S (2005). Mechanical strain opens connexin 43 hemichannels in osteocytes: a novel mechanism for the release of prostaglandin. *Molecular Biology of the Cell*.

[52] Romanello M, D’Andrea P (2001). Dual mechanism of intercellular communication in HOBIT osteoblastic cells: a role for gap-junctional hemichannels. *Journal of Bone and Mineral Research*.

[53] Saunders MM, You J, Trosko JE (2001). Gap junctions and fluid flow response in MC3T3-E1 cells. *The American Journal of Physiology*.

[54] Saunders MM, You J, Zhou Z (2003). Fluid flow-induced prostaglandin E2 response of osteoblastic ROS 17/2.8 cells is gap junction-mediated and independent of cytosolic calcium. *Bone*.

[55] Guilak F, Butler DL, Goldstein SA, Mooney DJ (2003). *Functional Tissue Engineering*.

[56] Farhadi J, Jaquiery C, Barbero A (2005). Differentiation-dependent up-regulation of BMP-2, TGF-*β*1, and VEGF expression by FGF-2 in human bone marrow stromal cells. *Plastic and Reconstructive Surgery*.

[57] Kanczler J, Oreffo R (2008). Osteogenesis and angiogenesis: the potential for engineering bone. *European Cells and Materials*.

[58] Huang Y-C, Kaigler D, Rice KG, Krebsbach PH, Mooney DJ (2005). Combined angiogenic and osteogenic factor delivery enhances bone marrow stromal cell—driven bone regeneration. *Journal of Bone and Mineral Research*.

